# Historic residential segregation impacts biodiversity data availability disparately across the tree of life

**DOI:** 10.1073/pnas.2416889122

**Published:** 2026-09-14

**Authors:** Diego Ellis-Soto, Melissa Chapman

**Affiliations:** ^a^ Department of Environmental Science Policy and Management, University of California, Berkeley, CA 94720; ^b^ Department of Ecology and Evolutionary Biology, Yale University, New Haven, CT 06511; ^c^ Department of Environmental Systems Science, ETH Zurich, Zurich 8001, Switzerland

**Keywords:** urban ecology, biodiversity sampling, environmental justice, Global Biodiversity Information Facility

## Abstract

Historical residential segregation policies have left a lasting impact on urban environments, greenspaces, and wildlife communities, creating socioeconomic heterogeneity and altering biota. However, the extent to which these policies continue to impact urban biodiversity remains unclear, especially across taxonomic groups and spatial scales. We explore how sampling and biodiversity metrics (sampling density, estimated completeness of sampling, and expected species richness) across taxonomic groups vary across historically redlined neighborhoods and relative to broader urban areas. To explore these patterns, we leverage nearly 60 million observations of amphibia, aves, fungi, insecta, mammalia, plantae, and reptilia collected between 2000 and 2020 within 145 Metropolitan Statistical Areas in the United States. We estimate significant differences in sampling density across redlined neighborhoods for all taxonomic groups, with the lowest values found in areas previously redlined. Estimated completeness of biodiversity inventory was low (average ~42% across all taxa) and varied significantly by Home Owners’ Loan Corporation (HOLC) grade for birds, mammals, and plants. Expected richness varied significantly by HOLC grade only for birds. Our findings highlight how differences in biodiversity sampling may not translate to differences in expected species richness patterns and suggest that applying insights obtained from certain taxonomic groups and extrapolating to multiple others may not be appropriate. Urban wildlife communities are not well-documented despite the explosion of digital information, and what is documented is known to be biased along gradients of housing segregation and multiple urban scales. These findings add evidence to suggest long-lasting effects of legacies of segregation on the natural world.

## Significance

Historic race-based zoning policies like redlining in the United States are associated with present day health, income, and environmental inequities. We quantify how redlining across 195 cities is related to key biodiversity metrics across all vertebrate, invertebrate, plant, and fungi species. We show that while more biodiversity records are consistently collected in nonredlined neighborhoods, this did not translate to differences in estimated species richness across redlining grades—except for birds. We further analyze these biodiversity facets across current metropolitan and urban areas. This work underpins how legacies of segregation, socioeconomic inequality, and current urban configurations may influence the availability and distribution of urban biodiversity data, potentially biasing our understanding on species communities, their food webs, and ultimately, conservation decisions.

Global projections suggest a 55 to 111% increase in urban area by 2100 [11 to 33 million hectares compared to 2015 (1)] and a vast increase in global population residing in these areas by 2050 (2). The last decade has seen an increased appreciation on the importance of urban biodiversity for promoting physical and psychological well-being of city residents (3). Cities are increasingly the spaces where human experiences with biodiversity occur (4) and also where a growing proportion of biodiversity face urban pressures. Urbanization therefore poses both opportunities and challenges for conservation (5); a challenge in the face of growing pressures and the complex disparate responses of species and taxa to urbanization but an opportunity in the wake of increasing investment and use of green infrastructure in urban planning and development (6, 7). As Lambert and Schell describe, “it is not hyperbolic to suggest that cities are situated as the literal and figurative frontlines of biodiversity conservation” (8).

Urban areas (UAs) represent complex systems strongly shaped by social and economic factors that are often characterized by social inequity. For example, socioeconomic disparities are associated with the spatial distribution of urban tree canopy cover, with higher income areas having more tree canopy, and marginalized communities having less (9–12). Tree canopy and urban green spaces provide crucial habitat for biodiversity, form the basis of more complex ecological communities, and shape urban food webs (12). Therefore, the socioeconomic partitioning of urban spaces is expected to shape multiple facets of urban biodiversity (13–15), albeit differently, and influence evolutionary processes and outcomes (13, 16–18).

Simultaneously, UAs are increasingly places of extensive biodiversity data collection, primarily through participatory-science and education initiatives on mobile phone data collection apps (19). For some species, particularly those commonly found in urban environments, volunteer-collected data far exceed records museum collections (20). There are biodiversity data disparities within and across countries: higher income countries have more information (21, 22), and higher income areas within high income countries have the most (23). These data biases skew our view of the natural world and mean that marginalized communities are often also data-poor (23–25), which may represent another form of environmental injustice and hinder conservation strategies.

Institutionalized racism is a major driver of social inequity, especially in cities (26). A spatial manifestation of institutionalized racism is housing segregation. One particularly well-known mapping of this housing segregation in cities across the United States was the Home Owners’ Loan Corporation (HOLC) in the mid to late 1930s, commonly known as Redlining. The HOLC, with input from local real estate actors, categorized neighborhoods (into “Grades”) based on a combination of housing stock (type, quality, age), favorable adjacent land uses such as parks and open space, proximity to transit, and the demographic and racial characteristics of the inhabitants. A-Graded areas were composed of primarily US-born White families living in new, single-family detached homes, were labeled “Best” and colored green on the maps. B-Graded or blue areas, labeled “Still Desirable” had older and/or denser housing stock. C-Graded or yellow areas, labeled “Definitely Declining” had more marginalized populations such as communities of color and/or immigrants. Finally, D-Graded or red areas, hence the name “redlining,” were labeled “Hazardous” and were composed of communities of color. It is important to note that the practices associated with redlining predate and postdate these maps. These practices are associated with covenants, codes, and restrictions (27); segregated newspaper advertisements for housing, among many others. These practices began in the early 1900s and continue today (28–31).

The urban ecology literature on redlining documents substantial disparities across neighborhood grades. Formerly A-Graded neighborhoods have more vegetation (32), more tree canopy (33–35), are cooler (36), and exhibit less noise pollution (37) than their formerly D-Graded counterparts. This means neighborhoods formerly comprised primarily of US-born Whites in single family detached homes are more hospitable today—for both people and other species—than areas classified as Hazardous, marked red on maps by HOLC, and denied home loans, because they were occupied by poorer communities of color and immigrants living in denser, older housing. In Baltimore, MD, street trees are larger and more species diverse in A-Graded areas than their formerly D-Graded counterparts (38), so they produce more ecosystem services and are more resilient to urban forest pathogens. Redlined neighborhoods in four cities in California have higher pollution burdens, less vegetation, hotter temperatures, and more noise pollution than A-Graded areas (39).

In addition to vegetation and street tree diversity, bird biodiversity data and species composition knowledge are significantly greater in A than D areas, with differences persisting even after controlling for population density, vegetation greenness, and protected open space (25). Moreover, field-based biodiversity assessments further showed that bird communities in Los Angeles vary across HOLC grades (40). For example forest birds and migratory birds were ~24% and ~17% more abundant, respectively, in formerly A- and B-Graded areas than C and D areas, while nonmigratory, introduced, and synanthropic (living near people, but nondomesticated species, like pigeons) dominated C-, and D-Graded areas (40). With discrepancies between volunteer bird data A- and D-Graded areas growing over time (25), there is pressing need to understand how housing segregation and urban biodiversity data relate to additional taxa. Early multitaxon work using the participatory science platform iNaturalist in four Californian cities shows that redlined areas have a lower number of insects, birds, and mammals species and that species composition varies by HOLC grade (41). However, how current urban configurations relate to historical legacies of segregation in terms of biodiversity facets remains largely unexplored. UAs are smaller census-defined footprints, capturing higher-density development blocks with at least 2,000 housing units or 5,000 people and often defined by land use intensity, whereas Metropolitan statistical areas (MSAs) reflect broader socioeconomic and commuter regions often spanning multiple counties and encompassing multiple habitats (https://www.census.gov).

This paper contributes to the ongoing efforts to address questions and test hypotheses about the ecological implications of housing segregation, specifically how race-based housing policies differentially impact multiple facets of urban biodiversity and biodiversity data availability (13). Synthesizing across platforms, the Global Biodiversity Information Facility (GBIF; https://www.gbif.org/) includes data from iNaturalist, eBird, other popular taxon-specific apps, as well as from participant node organizations composed of scientific research entities like universities and museums. In addition, this work further compares how biodiversity patterns across historic housing segregation compare to current designation of UAs. Building off and expanding upon prior research, we leverage 58,920,460 species observations from GBIF across metropolitan areas in the United States to assess how the amount of biodiversity information (sampling density), knowledge of species pools (completeness), and expected species richness varies by HOLC grades, UAs, and MSAs [Derived dataset GBIF.org (24 February 2025) Filtered export of GBIF occurrence data https://doi.org/10.15468/dd.4dbn34]. Sampling density answers the question about whether or not there are biodiversity data disparities today related to historic residential segregation. Completeness and expected species richness result from species accumulation curve extrapolations. These measures provide related estimations of unobserved biodiversity and, therefore, address the question of how present-day biodiversity data and biodiversity patterns relate to historic housing segregation.

This paper’s aims are therefore twofold: first, to understand biodiversity data disparities in UAs, and second to assess spatial variation in urban biodiversity. The HOLC classification system categorized residential neighborhoods in the mid-1930s, meaning ungraded areas were not yet urbanized or were urbanized but nonresidential land uses. Focusing only on graded areas excludes most of present-day American cities. By adding the nongraded UAs and MSAs, we provide two reference sets to contextualize HOLC neighborhoods in their larger urban contexts. This research thus broadens the taxa under investigation (*amphibia*, *aves*, *fungi*, *insecta*, *mammalia*, *plantae*, and *reptilia*) and uses a larger and more comprehensive set of species observations across multiple cities than previous related efforts (25, 41), while adding UA and MSA comparisons.

## Results

### Biodiversity Information Across HOLC Grades, UA, and MSAs.

Formerly A-Graded areas (areas deemed best and “safest” for investments) had significantly greater sampling density than D-Graded areas (“redlined” areas deemed hazardous) for all taxa except fungi (0.001 > *P* > 0.0001, *SI Appendix*, Fig. S1, unadjusted Wilcoxon rank sum tests without covariates, only assessing the raw number of biodiversity records). A-Graded areas had greater sampling density than either UA (*P* < 0.0001) or MSAs (*P* < 0.0001) for all nine taxonomic groups.

**Fig. 1.**
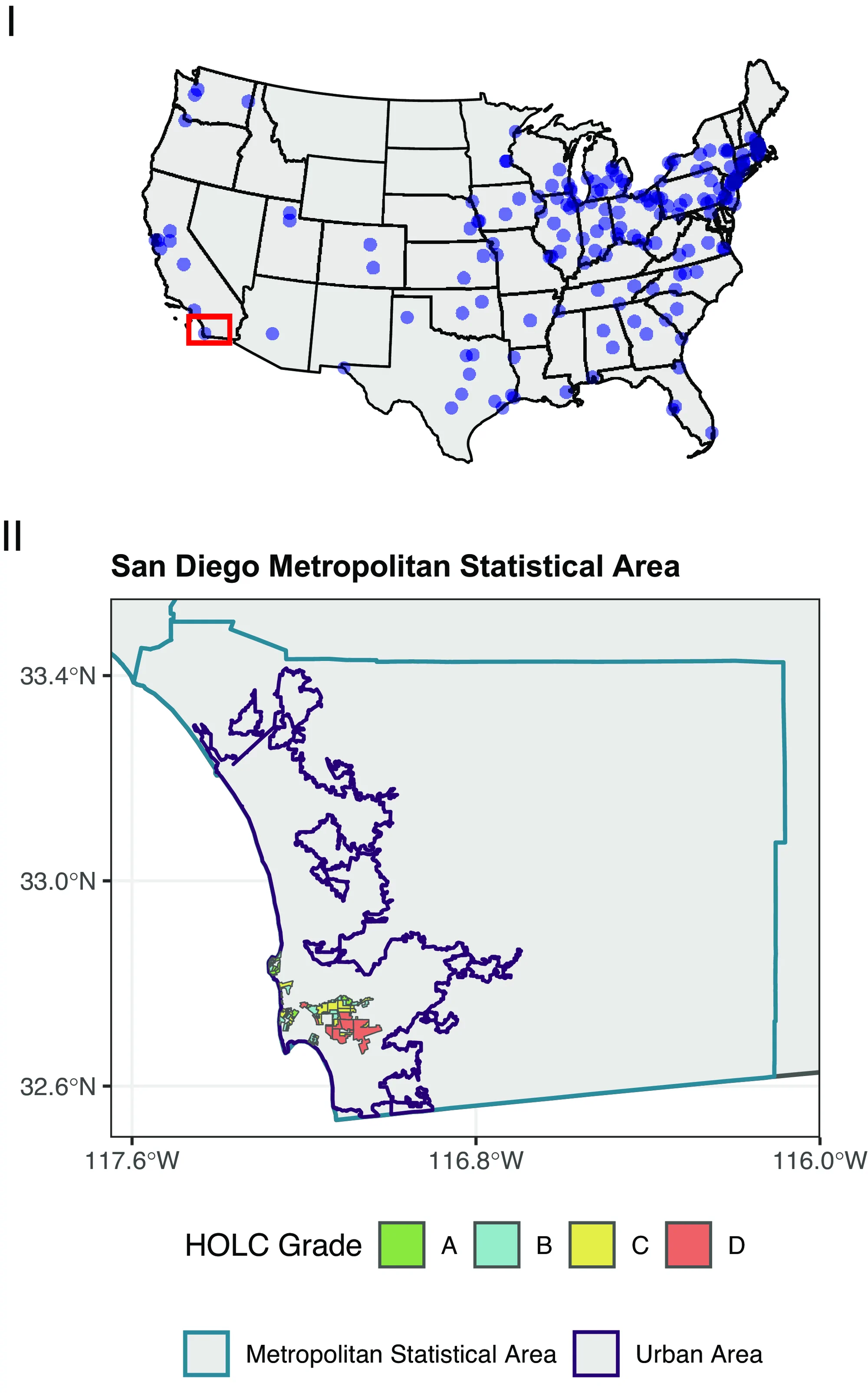
(*I*) (*Top* panel) Spatial extent of 195 cities assessed across the United States. (*II*) Spatial extent of our three urban categories of interest for the city of San Diego: MSAs (n = 145) included in the study. Within MSAs, UAs are smaller spatial units, as defined by the US Census Bureau. HOLC are typically nested within UAs but may not fully align with historical city boundaries. UAs on the other hand are located within MSAs, though there are a few instances where small parts of UA’s extend beyond an MSA boundary.

Completeness estimates from species accumulation curves represent how many species are expected to be present, if exhaustive sampling occurred. Completeness estimates were consistently low and did not vary by HOLC grade for amphibians, fungi (species or family), insects (family), mammals, or reptiles (*P* > 0.05) in the raw, unadjusted analyses. For birds, A-grade neighborhoods had greater completeness than B- (*P* < 0.05), C-, and D-graded (*P* < 0.001) neighborhoods (*SI Appendix*, Fig. S2). Conversely, completeness was greater in D-graded neighborhoods than A-graded neighborhoods for insects at the species level (*P* < 0.01) and among plants (*P* < 0.001, *SI Appendix*, Fig. S2). Overall, completeness was greatest in UAs and MSAs when compared to any HOLC-defined neighborhoods for all taxonomic groups (*P* < 0.0001, *SI Appendix*, Fig. S2). Expected richness did not vary by HOLC grade for taxonomic groups except for birds (*P* < 0.001), plants and reptiles (*P* < 0.05) (*SI Appendix*, Fig. S3). Expected richness was always greatest among MSAs (*P* < 0.0001) and UAs (0.001 > *P* > 0.0001) than for HOLC-Graded areas (*SI Appendix*, Fig. S3) (Fig. 1).

### Predictions of Biodiversity Information, Biodiversity Knowledge, and Species Richness Across HOLC Grades and UAs.

**Fig. 2.**
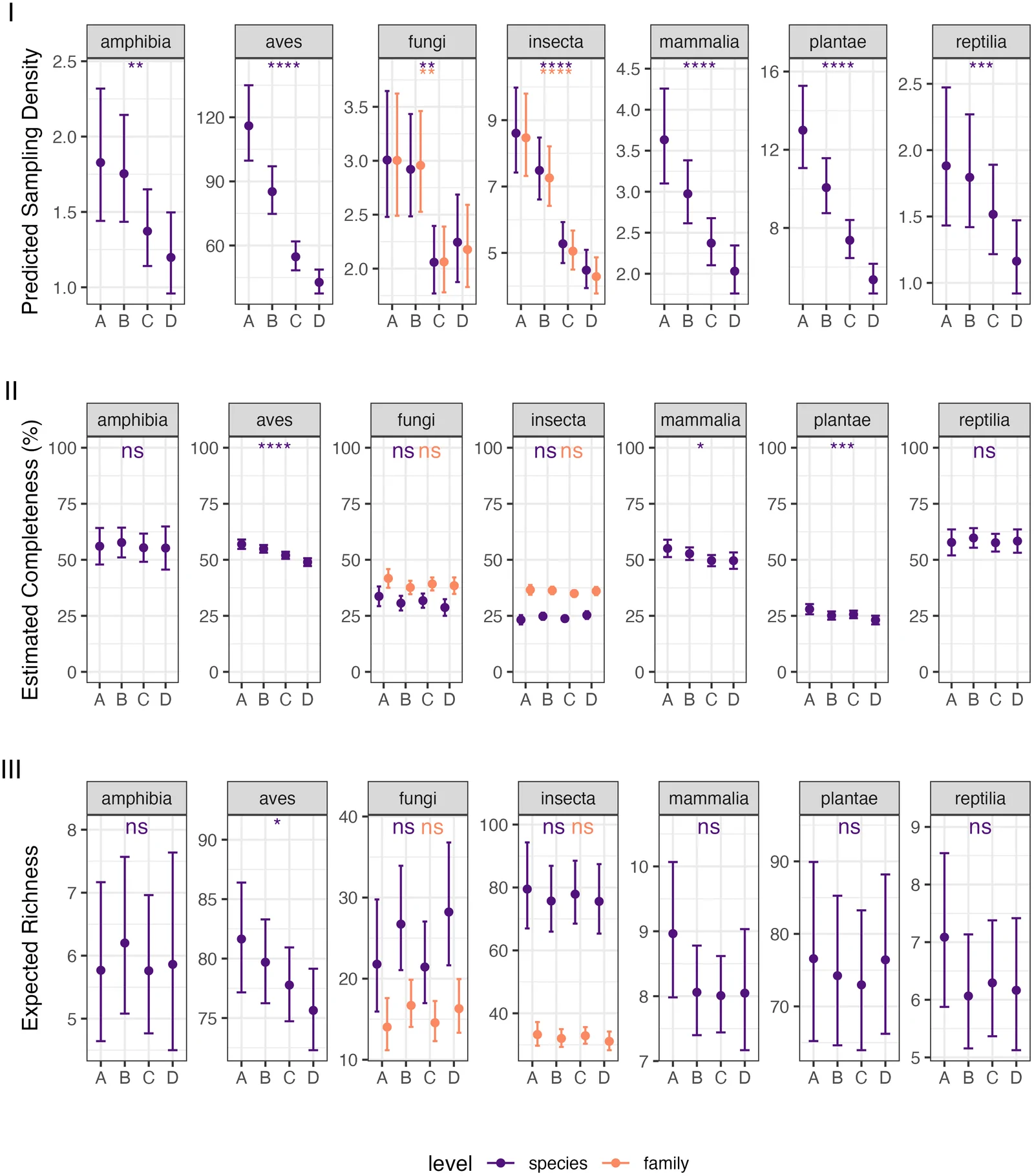
Model-adjusted predicted sampling density varies significantly across HOLC grade for all 9 taxonomic groups (*I*). Overall estimated completeness is low, and only varies for *aves*, *mammalia*, and *plantae* (*II*). The observed differences in sampling density and estimated completeness to not translate to differences by HOLC Grade for expected richness except for birds (*III*). Note the different vertical axes lengths.

Model predictions including covariates for population density, vegetation cover, protected open space, and water cover show significant differences (0.01 < *P* < 0.0001) in sampling density between formerly A-Graded neighborhoods and formerly D-Graded areas for all nine taxonomic groups (Fig. 2, *Top*). The amount of model-predicted biodiversity data varied widely by taxonomic group. For example, amphibian and reptile sampling density, though significantly different across A-Graded and D-Graded areas, were orders of magnitude lower than bird sampling density, regardless of HOLC grade.

Overall average model-predicted estimated completeness in formerly HOLC-defined neighborhoods was 41.7%, and lower for insects (mean estimated completeness = 24.3%), fungi (31.1%), and plants (25.4%)—the most species-rich taxonomic groups examined here (Fig. 2, *Middle*) across all HOLC grades. Model predictions showed significant differences in estimated completeness by HOLC grades A to D for birds (*P* < 0.0001), mammals (*P* < 0.05), and plants (*P* < 0.001), while the other six taxonomic groups were HOLC-invariant (*P* > 0.05). Birds were the only taxonomic group with significant differences in expected species richness across HOLC grades (Fig. 2, *Bottom*, *P* < 0.01).

## Discussion and Conclusions

In this study, we quantified how the race-based, housing segregation policy called redlining relates to the amount of biodiversity information and the number of expected species across multiple taxonomic groups encompassing nearly every facet of the tree of life. The goals were to both understand data collection biases and differences in urban biodiversity across varied neighborhoods. Despite prior research on redlining and biodiversity in smaller geographic regions (41) or with limited taxonomic focus (25, 40), it remained unclear whether data disparities reflected a general pattern across multiple taxa and cities or if these patterns were heterogeneous. Further, we explicitly integrated multiple urban scales—HOLC neighborhoods, UAs, and MSAs—to contextualize patterns of urban biodiversity data alongside historical housing segregation

Sampling density was greater in formerly A-Graded neighborhoods than formerly D-Graded neighborhoods for all taxonomic groups examined (Fig. 2). Moreover, sampling density is greater in HOLC neighborhoods than their encompassing UAs and metropolitan regions, while the reverse was true for estimated completeness and expected richness. Few prior investigations have included nongraded comparisons (40), despite calls to do so (31). These patterns are unsurprising given differences in population density across these places, which reduce sampling density among the larger and less population dense spatial units, reflecting the amount of data in areas with higher populations. It remains unclear why people choose to record biodiversity data in formerly A-Graded areas compared to formerly D-Graded areas. One explanation is that there is more green space and tree canopy in A than D-areas (32–35), making these more attractive places to travel to and sample records. Another explanation is that people in D-Graded neighborhoods are observing biodiversity but do not have the time or capacity to report or are unaware of public science platforms. Or people may collect observations in D-Graded areas and not report their data digitally. Those observing and reporting urban biodiversity may already predominantly reside disproportionately in formerly A-Graded areas. The combination of GBIF and HOLC polygons alone does not let us arbitrate between these rival and complementary explanations.

While sampling density differed across HOLC grades for all taxonomic groups, differences in regression-adjusted estimated completeness of biodiversity inventory were only found in birds, mammals, and plants. Differences in expected species richness across HOLC grades were unique to birds. The birdwatching community may promote collecting and sharing data more than for other taxa, and mammal identification is relatively easier. Plants are immobile, very species rich, and can be relatively challenging to identify based on their phenotype, while there are few urban mammal species. Insects and fungus identification is frequently even more challenging, and reptiles and amphibians are relatively rarer, especially in UAs. Additionally, amphibians have notoriously low detection probabilities, even if present, so they might be present and overlooked (42). These attributes may contribute to our documented taxon-specific findings. Future studies may consider quantifying species abundances or densities with colocated measurements across taxonomic groups. This may allow for answering questions about whether communities and wildlife food webs vary by race-based policies, as proposed by Schell and colleagues in 2020 (13).

### More Sampling Density in A-Grade in All Taxa.

Our findings that all taxonomic groups had higher sampling density in HOLC-A graded than in D-Graded areas, corroborate the relationships found among birds in prior empirical research (25) and supporting expectations (13). This evidence further suggests how formerly redlined areas have not only fewer environmental amenities today (32–34), greater pollution loads (39), but also less information across nearly every facet of biodiversity. These differences persisted after accounting for human population density, vegetation productivity, protected and accessible open space, and water cover. Similar findings were observed in four Californian cities across six clades, using only iNaturalist data, effectively a subset of GBIF (41). The data disparities found in the larger and more comprehensive GBIF data used here, and across a wider range of taxonomic groups, are reflected within a subset of participatory science platforms, when examining a smaller subset of species in a specific geographic location.

### Taxonomic Groups Differ in Data Availability and Survey Completeness.

Completeness estimates of biodiversity data varied across taxa. Fungi, insects, and plants had the lowest estimated completeness, yet are the most species-rich taxonomic groups on earth. Of the observations analyzed here, 87.6% were birds, 7.37% plants, 3.16% insects, and the remaining ~2% were fungus, mammals, reptiles, and amphibians combined. To date, most urban ecology research has focused on birds and vascular plants (43), with invertebrates being among the least studied group (44). In addition, groups such as amphibians and reptiles remain even less-studied, despite being the vertebrate groups facing the highest rates of extinctions in the Anthropocene (45, 46). The taxonomic bias in urban ecology research remains a crucial knowledge gap, as identified by studies calling to include more taxonomic groups (47). Using estimated completeness, we show how the collective information on urban biodiversity differs across taxonomic groups. Specifically, we show higher survey completeness for birds, mammals, amphibians, and reptiles than plants, fungi, and insects. Low levels of completeness in plants, fungi, and insects likely do not accurately reflect species richness patterns, as these groups are species rich when compared to vertebrates and sampling density was relatively low—it is therefore challenging to disentangle these relationships. More comparative studies across multiple taxa, geographic areas, and over time might be considered a research priority in urban ecology (43).

We did not observe significant differences in expected species richness by HOLC grade in any taxa except for birds (Fig. 2). For example, our models predicted similar expected species richness across HOLC grades for birds than for insects and plants, despite there being orders of magnitude more described insect and plant species across the United States than bird species. For example, there are ~1,150 bird species in the United States of America, but ~91,000 insect species and 16,670 vascular plant species (48–50). Our findings therefore may be reasonably indicative of the low sampling completeness among HOLC grades and the difficulty accurately identifying some species without molecular biology in plants, fungi, and insects when compared to birds, mammals, reptiles and amphibians. Low sampling density, especially for species-rich groups, translates into low survey completeness and unrealistically low expected richness, severely limiting ecological inferences about actual community assemblages when using these types of data. Again, more extensive and targeted, local field, possibly with taxonomic experts, sampling may prove pivotal to better understand current urban biodiversity patterns.

### Biodiversity Facets Across Today’s Cities: UAs and Metropolitan State Areas.

Our comparison with UAs and MSAs reveals that broader UAs—while showing lower per-area sampling densities than HOLC-graded neighborhoods—tend to exhibit higher estimates of completeness and expected richness overall. This suggests that relatively large administrative units encompass more diverse habitats that single HOLC grades in cities and potentially host many species that go undersampled at finer neighborhood scales

### Implications.

Taken together, our results suggest against extrapolating patterns and social drivers of data availability or biodiversity metrics from one taxonomic group to another, particularly when making inferences on invertebrates, plants or fungi based on vertebrate biodiversity patterns. Sampling density, completeness, and bird richness are not representative of other taxonomic groups in urban environments when using these synthesized observations primarily from participatory science data. Biodiversity data from birds in particular may be distinct from other taxa in several ways: a) birds have significantly more observations than other taxa, b) the spatial distribution of their biodiversity records and expected species richness is matched by the rank-order of the HOLC’s neighborhood ranking system, and c) birds are a highly mobile taxon. The rise of participatory science campaigns such as eBird and iNaturalist have led to a rapid and steady increase in the collection of such biodiversity data across the world, but participation and uptake are primarily by well-educated, white and affluent adults (51, 52). Future work could analyze the demographic profiles of relatively small census geographies like tracts or block groups in association with GBIF data to identify how present-day socioeconomic conditions relate to sampling density and urban biodiversity (23, 24). Concurrently, more research examining the socioeconomic and demographic composition at the individual observer level may reveal patterns and trends by taxonomic and social groups.

While the increasing use of crowdsourced, geolocated bird data in scientific studies and conservation decisions have led to policy recommendations in urban environments (53), observed trends of bird biodiversity may not necessarily reflect other taxonomic groups of vertebrates, invertebrates, and plants. In an era of ambitious global conservation, careful consideration for how data availability across space impacts ecological inference differently across taxonomic groups, and impacts downstream uses is warranted (22). It is also worth considering how these monitoring initiatives themselves impact nature. Furthermore, nature friendly architectural and urban planning practices increasingly consider the disparate needs of species based on their life-history traits and biology (54, 55).

Future work may provide more in-depth exploration into specific facets of biodiversity utilizing other biodiversity data repositories, such as the BIEN database for plant-specific analysis (56). Ultimately, more long-term and locally collected field data are likely needed to understand whether and how species communities and food webs are impacted by socioeconomic conditions within and across cities. Moreover, how those relationships themselves vary with race-based housing segregation remains less clear.

We are just beginning to understand how past and present practices of segregation and socioeconomic inequality have left (and are leaving) a lasting impact on the environment, urban wildlife communities, food webs, and their evolution (8, 13). Understanding the implications of these human dimensions could be critical for the equitable planning and execution of ambitious conservation policy and sustainability initiatives from local to national levels that integrate nature’s contribution to people (57). Ecologists increasingly incorporate multiple aspects of human activities into biodiversity studies—from movement, to biproducts such as nightlights, roads, and population density and land use change (58). Yet, socioeconomic disparities in biodiversity data are an often overlooked, but critical dimension to consider when leveraging these data for ecological insights or decision making (22). While historic redlining was just one of many housing segregation practices, future research could incorporate Urban Renewal project locations (https://dsl.richmond.edu/panorama/renewal/#view=-7726.48/ 3679.22/11.13&viz=map&city=baltimoreMD&loc=13/39.2972/-76.5880) as well as additional city specific practices. With urbanization poised to intensify and the human-wildlife overlap growing worldwide in the future, it becomes critical to better understand associated social and environmental challenges at the wildlands–urban interface (59). As such sampling facets of biodiversity across such exurban fringes while considering socio-economic gradients driving such access to information will become critical for more environmental just urban planning and design of nature friendly cities in the future.

This work provides strong evidence of differences where we collect information of biodiversity across multiple taxonomic groups across large spatial extents, filling important knowledge gaps in urban ecology and environmental justice research. Future researchers may consider exploring how functional and phylogenetic diversity of these taxonomic groups differs across urban environments, providing a more ecologically rich context on how species communities vary within and across past, present, and future UAs. Future researchers may consider including more measurements on where segregationist policies shaped the built and social environments, which in turn affects the ecological contexts for other species.

## Materials and Methods

### Study Area.

We obtained biodiversity information for 195 cities with existing digitized HOLC polygons at the time of our analysis. In order to include nongraded areas as a reference, two Census-defined units were used: UA and MSAs. UAs are the smaller spatial unit among the two and created by aggregating Census blocks that have 5,000 people or 2,000 housing units. Furthermore, UAs are defined through their makeup of population as well as land use intensity and thus vary substantially across cities. MSA’s are commutable area designations and encompass larger areas spanning multiple land use and habitat types. MSA involve aggregations of counties with at least 50,000 people. UA and MSA boundaries were accessed via the “get_acs” function in the tidycensus package (60). Every MSA that contained digitized HOLC polygons that contained with GBIF data (n = 8,207) were included. The result was 145 MSAs, 147 UAs contained within 38 states, within 195 cities which had spatial information on redlined areas. When calculating the sampling density, completeness, and expected richness, HOLC polygons were omitted from their containing UAs and MSAs to avoid double-counting their biodiversity observations.

### Biodiversity Data.

Biodiversity observations came from the GBIF (https://www.gbif.org/), via the “gbif_remote” function in the gbifdb R package (61). GBIF synthesizes disparate sources of biodiversity data from repositories ranging from participatory science apps to museum collections. Observations were filtered to observations containing georeferenced records collected between 2000 and 2020, that were not fossil specimens or material. The total number of observations (n = 58,920,460) per taxon downloaded were amphibia (n = 131,585), aves (n = 51,590,588), fungi (n = 577,360), insecta (n = 1,864,414), mammalia (n = 224,351), plantae (n = 4,342,105), reptilia (n = 190,057). HOLC polygons were obtained from the University of Richmond’s Mapping Inequality Project (62) via https://dsl.richmond.edu/panorama/redlining/static/fullDownload.geojson on December 8, 2022.

The three dependent variables analyzed were sampling density, completeness, and expected richness. Sampling density was calculated as the number of observations records per square kilometer. Completeness (%) and expected species richness were calculated using species accumulation curves via the “KnowBPolygon” function in the KnowBR package (63). Completeness represents the percentage of all species estimated to be present given the observed GBIF observations within a spatial unit (HOLC polygon, UA, or MSA). Expected richness was calculated by extrapolating species accumulation curves (63).

### Covariates.

In regression analyses, each of the dependent variables was modeled as a function of population density, vegetation cover, protected open space, and water cover. Prior research on birds and HOLC polygons has found significant relationship between human population density, NDVI, and open space with sampling density and percent estimated completeness (25). Additionally, it could be expected that places with more people could be more likely to have participatory science-collected biodiversity data since more potential observers are present. Population counts for HOLC polygons were interpolated using an area-weighted method, where the population was attributed by percent of polygon overlap (64) and year 2019 Census block groups accessed via the “get_acs” function in the tidycensus package (60). Normalized Difference Vegetation Index (NDVI) was computed using the mean of the average monthly MODIS (250 m) data from 2015 to 2019. NDVI captures photosynthetically active plants and was included as a vegetation measure. The percent cover of protected open space was included because observers are likely to use parks and open space to collect data. We used a version of USGS’ Parks and Protected Areas Database of the United States (PAD-US) that was augmented to include accessible and recreational lands (PAD-US-AR), which is a more accurate and comprehensive representation of open space (65). Spatial water data came from the US Fish and Wildlife Service’s National Wetlands Inventory (https://www.fws.gov/program/national-wetlands-inventory/download-state-wetlands-data).

### Statistical Analyses.

Two sets of statistical analyses were performed: 1) an unadjusted examination of each dependent variable for each taxonomic group by HOLC grade, UA, and MSA categories; and 2) regression analyses excluding UA- and MSA-observations but including continuous covariates. In both cases, sampling density and expected richness were log-transformed to approximate normal distributions. In the first set of analyses, each outcome in the A-Graded polygons was analyzed against the B-, C-, D-Graded UA’s and MSA’s values in a series of 5 pair-wise Wilcoxon rank sum tests. Not all possible pairwise tests were performed, rather the endmember was compared against each other value; A serves as a reference and all other values are relative. *SI Appendix*, Figs. S1–S3 show the entire distributions.

Regression analysis incorporated all HOLC polygons but omitted the UAs and MSAs. This is because UA and MSA represent large geographic areas with high levels of internal heterogeneity, making interpretations difficult. Within an MSA, the mean NDVI does not adequately represent the internal distribution which may have values of zero and one. A mean of 0.5 would not faithfully characterize the region in social or ecological terms. Instead, each of the three dependent variables was analyzed for each of the nine taxonomic groups with three different regression model specifications. The first specification was the outcome as a function of the HOLC grade alone. This linear model is a baseline, simple model. The second specification added a random intercept for unobserved variability associated with each MSA. The third and most complex model adds continuous covariates to the mixed model to control for population density (people per km^2^), mean NDVI (a measure of vegetation greenness), protected accessible open space (%, from PAD-US-AR), and water cover (%, from the National Wetlands Inventory). The second and third specifications were fit with the lme4 package (66) in R. Per dependent variable and taxonomic group, the AIC minimization criteria was used to find the best fitting and parsimonious model among the three specifications. Model predictions were then derived with the “ggpredict” function and pairwise significance testing was applied using the “hypothesis_test” functions in the ggeffects package (67).

## Supplementary Material

Appendix 01 (PDF)

## Data Availability

Underlying raw data are available on GBIF under https://www.gbif.org/derivedDataset/10.15468/dd.4dbn34 (68), the summarized analysis-ready data, and the R scripts for curating, compiling and conducting the final analyses are available in https://github.com/milliechapman/biodiversity_in_a_concrete_jungle (69). All other data are included in the manuscript and/or *SI Appendix*.
