# Supplementary material for: Historic residential segregation impacts biodiversity data availability disparately across the tree of life
Source: Proc Natl Acad Sci U S A. 2026 Sep 14;123(38):e2416889122. doi: 10.1073/pnas.2416889122 (PMC13598210; doi:10.1073/pnas.2416889122)
Supplement: Supplementary file 1 [file pnas.2416889122.sapp.pdf]

Historic residential segregation impacts biodiversity data availability disparately across the tree of life

Diego Ellis-Soto[1,2], Melissa Chapman[3]

1 Department of Environmental Science Policy and Management, University of California, Berkeley, CA, USA ORCID: 0000-0003-4766-021X

2 Department of Ecology and Evolutionary Biology, Yale University, New Haven, USA

3 Department of Environmental Systems Science, ETH Zurich, Zurich ORCID: 0000-0002-1377-1539

Diego Ellis Soto, Melissa Chapman
Email: diego.ellissoto@berkeley.edu; ; melissa.chapman@usys.ethz.ch

Supplementary Material

Table S1. Descriptive Statistics of biodiversity metrics and model coefficients across taxonomic groups.

Characteristic	amphibia: species, N = 503[1]	aves: species, N = 7,717[1]	fungi: family, N = 1,374[1]	fungi: species, N = 1,341[1]	insecta: family, N = 4,473[1]	insecta: species, N = 4,432[1]	mammalia: species, N = 1,943[1]	plantae: species, N = 5,774[1]	reptilia: species, N = 861[1]
Sampling Density (log)	0.55 (-0.16, 1.23)	4.38 (3.16, 5.49)	1.04 (0.31, 1.87)	1.05 (0.31, 1.88)	1.93 (1.12, 2.86)	1.93 (1.12, 2.88)	1.06 (0.36, 1.83)	2.26 (1.40, 3.31)	1.06 (0.26, 1.85)
Estimated Completeness	56 (40, 69)	58 (38, 74)	39 (28, 49)	29 (18, 40)	36 (24, 48)	23 (15, 35)	48 (40, 65)	22 (13, 39)	62 (42, 76)
Unknown	360	1,327	842	910	1,945	2,407	1,054	2,930	460
Expected Richness (log)	1.70 (1.26, 2.12)	4.44 (4.06, 4.81)	2.88 (2.13, 3.49)	3.47 (2.63, 4.19)	3.64 (3.06, 4.09)	4.47 (3.78, 5.07)	2.01 (1.60, 2.53)	4.72 (4.04, 5.32)	1.90 (1.33, 2.26)
Unknown	360	1,327	842	910	1,945	2,407	1,054	2,930	460
HOLC Grade									
A	95 (19%)	929 (12%)	214 (16%)	211 (16%)	555 (12%)	552 (12%)	274 (14%)	726 (13%)	132 (15%)
B	140 (28%)	2,009 (26%)	397 (29%)	383 (29%)	1,208 (27%)	1,198 (27%)	578 (30%)	1,530 (26%)	244 (28%)
C	177 (35%)	3,010 (39%)	524 (38%)	514 (38%)	1,796 (40%)	1,782 (40%)	761 (39%)	2,325 (40%)	305 (35%)
D	91 (18%)	1,769 (23%)	239 (17%)	233 (17%)	914 (20%)	900 (20%)	330 (17%)	1,193 (21%)	180 (21%)
Population / km^2	647 (313, 1,259)	1,324 (659, 2,694)	852 (410, 1,650)	845 (408, 1,636)	1,012 (513, 1,991)	1,009 (513, 1,978)	855 (438, 1,667)	1,137 (578, 2,283)	798 (389, 1,578)

Characteristic	amphibia: species, N = 503[1]	aves: species, N = 7,717[1]	fungi: family, N = 1,374[1]	fungi: species, N = 1,341[1]	insecta: family, N = 4,473[1]	insecta: species, N = 4,432[1]	mammalia: species, N = 1,943[1]	plantae: species, N = 5,774[1]	reptilia: species, N = 861[1]
NDVI (mean)	0.43 (0.35, 0.50)	0.41 (0.32, 0.48)	0.40 (0.29, 0.49)	0.40 (0.29, 0.49)	0.40 (0.30, 0.47)	0.39 (0.30, 0.47)	0.38 (0.28, 0.47)	0.40 (0.31, 0.48)	0.37 (0.28, 0.46)
Protected Open, Accessible Space (%)	2.5 (0.9, 7.3)	1.5 (0.1, 4.4)	2.6 (0.9, 6.4)	2.6 (0.9, 6.4)	1.9 (0.5, 5.0)	1.9 (0.5, 5.0)	2.4 (0.8, 5.6)	1.8 (0.4, 4.8)	1.7 (0.4, 4.8)
Water (%)	0.19 (0.00, 0.97)	0.00 (0.00, 0.67)	0.02 (0.00, 0.67)	0.02 (0.00, 0.67)	0.00 (0.00, 0.62)	0.00 (0.00, 0.62)	0.01 (0.00, 0.69)	0.00 (0.00, 0.65)	0.09 (0.00, 0.69)

[1] Median (IQR); n (%)

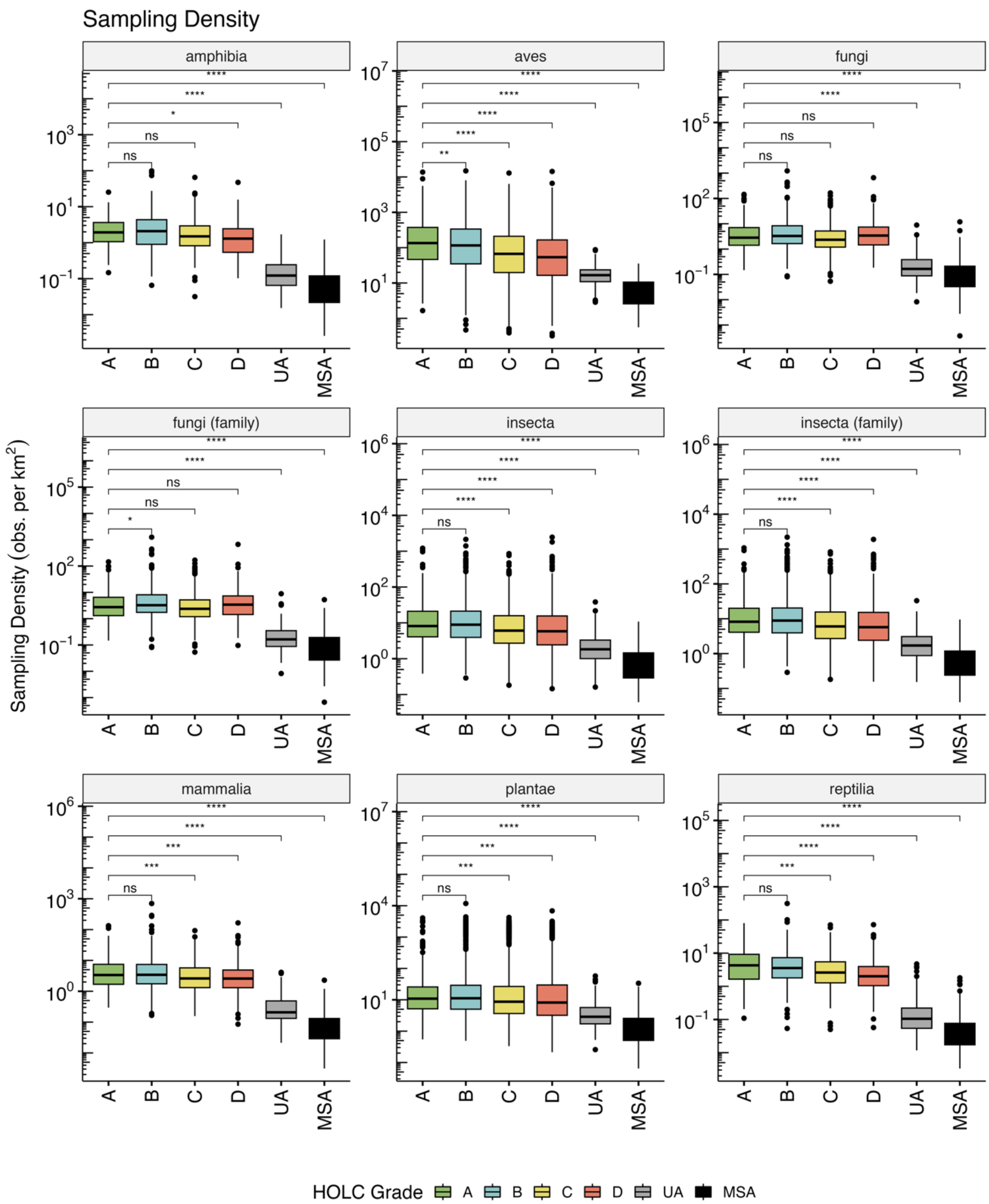

Figure S1. Sampling Density (the number of volunteered-collected observations per area) vary by Home Owners Loan Corporation neighborhoods, with areas formerly A-Graded having more biodiversity information than areas formerly D-Graded for all taxon except for fungi at both species and family levels. Sampling density in HOLC polygons, was greater than their encompassing Census-defined Urban Areas (UA) and Metropolitan Statistical Areas (MSA).

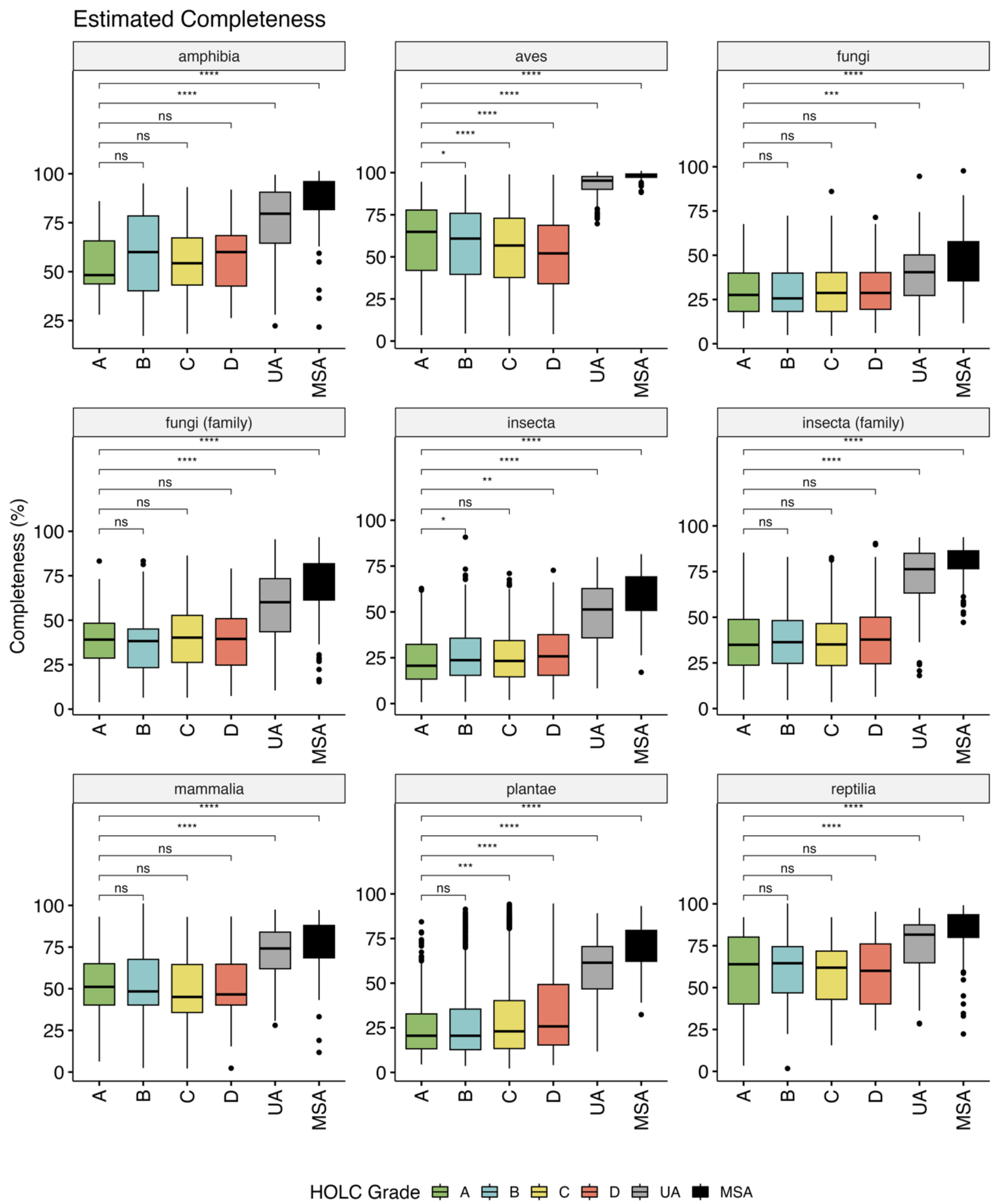

Figure S2. When sampling density is used to estimate percent completeness, few statistically significant differences emerged. A-Graded areas have more complete biodiversity data than D-Graded areas for birds (*aves*), but the association is reversed for insects (at species and family levels) and for plants. The percent completeness is relatively low overall, and especially for insects, fungus, and plants. Despite fewer observations per area for sampling density, the percent completeness is greater in Urban Areas and Metropolitan Statistical Areas than HOLC polygons, owing to their larger size.

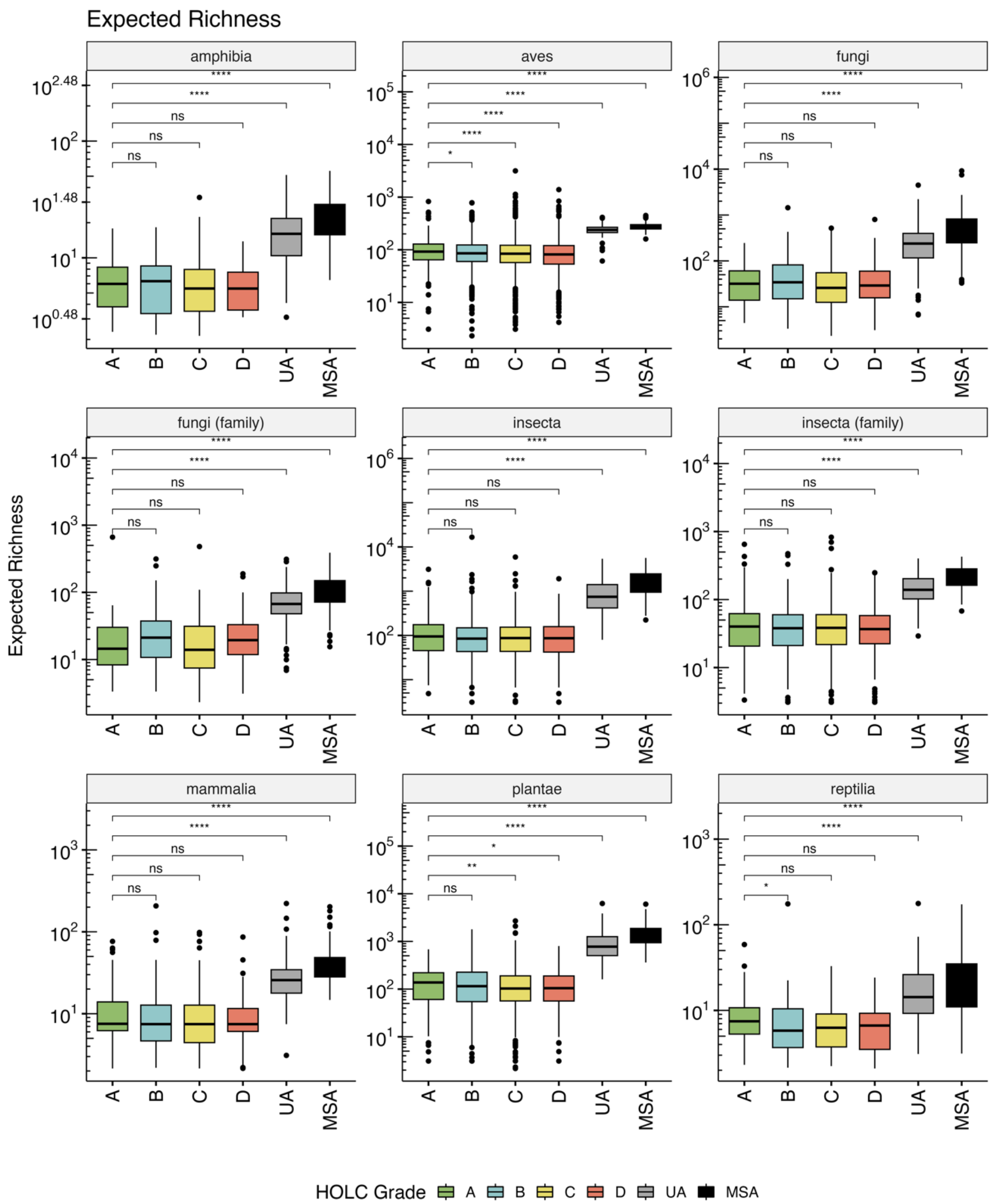

Figure S3. Expected species richness patterns across taxonomic groups. Only *aves* and *plantae* expected richness vary by HOLC grade, the other taxon are invariant to the neighborhood classification system.
